# General practitioners’ perceptions on opportunistic single-time point screening for atrial fibrillation: A European quantitative survey

**DOI:** 10.3389/fcvm.2023.1112561

**Published:** 2023-02-15

**Authors:** Paulien Vermunicht, Mihaela Grecu, Jean-Claude Deharo, Claire M. Buckley, Elena Palà, Georges H. Mairesse, Michal M. Farkowski, Marco Bergonti, Helmut Pürerfellner, Coral L. Hanson, Lis Neubeck, Ben Freedman, Henning Witt, Mellanie T. Hills, Jenny Lund, Katrina Giskes, Daniel Engler, Renate B. Schnabel, Hein Heidbuchel, Lien Desteghe, for the AFFECT-EU investigators

**Affiliations:** ^1^Department of Cardiology, Antwerp University Hospital, Antwerp, Belgium; ^2^Research Group Cardiovascular Diseases, University of Antwerp, Antwerp, Belgium; ^3^Electrophysiology Department, Cardiovascular Diseases Institute, Iasi, Romania; ^4^Assistance Publique − Hôpitaux de Marseille and Aix Marseille Université, C2VN, Marseille, France; ^5^School of Public Health, University College Cork, Cork, Ireland; ^6^Neurovascular Research Laboratory, Vall d’Hebron Institute of Research (VHIR) - Universitat Autónoma de Barcelona, Barcelona, Spain; ^7^Cliniques du Sud Luxembourg, Arlon, Belgium; ^8^II Department of Heart Arrhythmia, National Institute of Cardiology, Warszawa, Poland; ^9^Department of Cardiology, Ordensklinikum Linz Elisabethinen, Linz, Austria; ^10^School of Health and Social Care, Edinburgh Napier University, Edinburgh, United Kingdom; ^11^Heart Research Institute, The University of Sydney, Sydney, NSW, Australia; ^12^Charles Perkins Centre and Concord Hospital Cardiology, The University of Sydney, Sydney, NSW, Australia; ^13^Pfizer Pharma GmbH, Berlin, Germany; ^14^StopAfib.org, Dallas, TX, United States; ^15^Primary Care Unit, Department of Public Health and Primary Care, University of Cambridge, Cambridge, United Kingdom; ^16^Department of General Practice, School of Medicine, University of Notre Dame, Sydney, NSW, Australia; ^17^Department of Cardiology, University Heart and Vascular Center Hamburg Eppendorf, Hamburg, Germany; ^18^German Center for Cardiovascular Research (DZHK), Partner Site Hamburg/Kiel/Lübeck, Hamburg, Germany; ^19^Faculty of Medicine and Life Science, Hasselt University, Hasselt, Belgium; ^20^Heart Center Hasselt, Jessa Hospital, Hasselt, Belgium

**Keywords:** atrial fibrillation, screening, AFFECT-EU, survey, general practitioners

## Abstract

**Background:**

There is no clear guidance on how to implement opportunistic atrial fibrillation (AF) screening in daily clinical practice.

**Objectives:**

This study evaluated the perception of general practitioners (GPs) about value and practicalities of implementing screening for AF, focusing on opportunistic single-time point screening with a single-lead electrocardiogram (ECG) device.

**Methods:**

A descriptive cross-sectional study was conducted with a survey developed to assess overall perception concerning AF screening, feasibility of opportunistic single-lead ECG screening and implementation requirements and barriers.

**Results:**

A total of 659 responses were collected (36.1% Eastern, 33.4% Western, 12.1% Southern, 10.0% Northern Europe, 8.3% United Kingdom & Ireland). The perceived need for standardized AF screening was rated as 82.7 on a scale from 0 to 100. The vast majority (88.0%) indicated no AF screening program is established in their region. Three out of four GPs (72.1%, lowest in Eastern and Southern Europe) were equipped with a 12-lead ECG, while a single-lead ECG was less common (10.8%, highest in United Kingdom & Ireland). Three in five GPs (59.3%) feel confident ruling out AF on a single-lead ECG strip. Assistance through more education (28.7%) and a tele-healthcare service offering advice on ambiguous tracings (25.2%) would be helpful. Preferred strategies to overcome barriers like insufficient (qualified) staff, included integrating AF screening with other healthcare programs (24.9%) and algorithms to identify patients most suitable for AF screening (24.3%).

**Conclusion:**

GPs perceive a strong need for a standardized AF screening approach. Additional resources may be required to have it widely adopted into clinical practice.

## 1. Introduction

The prevalence of atrial fibrillation (AF), currently 2–4%, will increase further worldwide in the next decades (1). Although AF episodes are commonly accompanied by symptoms, episodes occur asymptomatically in one patient in three (2). Nevertheless, asymptomatic patients are at increased risk of stroke, other cardiovascular complications, and death, comparable to symptomatic patients. With timely AF diagnosis, oral anticoagulant therapy can reduce the stroke risk with >60% (3). The increasing prevalence, the risk of asymptomatic AF and the increasing availability of AF detection tools, have fueled international interest in AF screening methods (4).

The European Society of Cardiology (ESC) AF guidelines recommend opportunistic AF screening by pulse taking or electrocardiogram (ECG) rhythm strip in patients ≥65 years (5). Despite these recommendations, the optimal setting with the highest efficiency for implementation in daily clinical practice remains uncertain among experts (4).

A prior European qualitative study of the AFFECT-EU project was conducted in which regulators and healthcare professionals with expertise in (AF) screening pathways were interviewed on the feasibility and challenges of implementing different AF screening scenarios. The experts considered single-time point opportunistic screening in primary care using single-lead ECG devices as the most feasible approach to screen for AF (6). However, information on the opinion of general practitioners (GPs) remains largely unknown.

The aim of this study was to evaluate the overall perception of GPs in Europe concerning the value and practicality of implementing AF screening in daily clinical practice, focusing on opportunistic single-time point screening with a single-lead ECG device. The results provide insight into the general and region-specific opportunities and obstacles for implementation of AF screening at scale. In addition, this study identified the preferred strategies to overcome screening-related barriers.

## 2. Methods

### 2.1. Survey

A European, descriptive, cross-sectional study was conducted with a specifically developed survey (Supplementary annex 1). The survey was designed by the coordinating investigators and consisted of four different parts: (i) general demographic characteristics of the respondents, (ii) overall availability and perception of screening approaches for AF and other conditions, (iii) feasibility of opportunistic single-time point screening with a single-lead ECG device, and (iv) possible requirements and barriers for the implementation of AF screening. The survey was validated for its content (Supplementary annex 2) and took approximately 5–7 min to complete.

### 2.2. Target audience

Given the results of the previously performed qualitative study, the target audience to complete this survey consisted of GPs (6). Nevertheless, other relevant primary healthcare professionals, such as nurses and scientists (*n* = 38), were not excluded. In contrast, some responses of secondary care based physicians, i.e., cardiologists and geriatricians (*n* = 10), were excluded from analysis.

### 2.3. Dissemination

The survey was implemented electronically *via* Qualtrics (Provo, Utah, United States) and could be accessed *via* a weblink. The dissemination was performed by firstly contacting European GP organizations. In a second phase, also personal GP networks within the AFFECT-EU consortium and beyond were contacted. To reduce the possible impact of language barriers, the survey was made available in Dutch, German, Spanish, French, Italian, Polish and Romanian, in addition to English (language choice was made before respondents gave their consent to participate). Responses were collected between July 2021 and May 2022. Ethics approval was granted by Ethik-Kommission der Ärztekammer Hamburg.

### 2.4. Statistics

The response data were downloaded in Microsoft Excel and further analyzes occurred in SPSS (version 28.0, IBM, NY, United States). Descriptive statistics were used to analyze the responses. Countries were classified into European subregions using the United Nations (UN) Geoscheme. Although the UN Geoscheme is the most commonly used scheme to divide European countries into geographical regions, it may not reflect the differences between health care systems in countries. According to the scheme, United Kingdom & Ireland are counted as part of Northern Europe. However, due to known differences in health system, United Kingdom & Ireland were considered a separate sub-region in this study. To check whether two categorical variables were associated, the chi-square test was applied. The Kruskal Wallis test assessed whether the medians of different European regions were equal. Results were considered significant for *p*-values <0.05.

## 3. Results

### 3.1. Demography of the respondents

**Figure 1:**
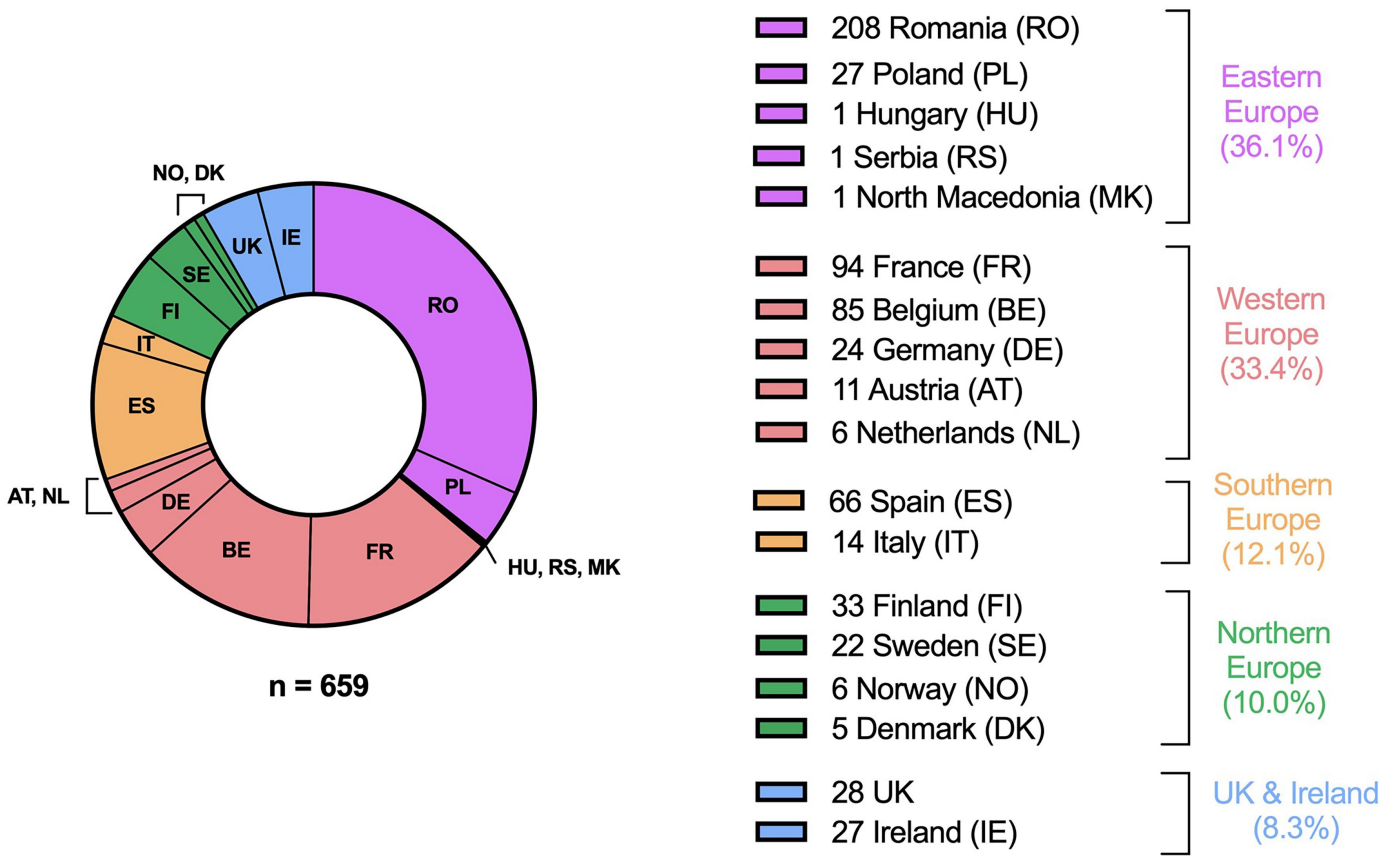
Origin of the respondents. Country groups are based on The United Nations Geoscheme, except for the UK and Ireland which were considered a separate sub-region due to the difference in health system.

A total of 596 respondents completed all four parts of the survey. A further 16 and 47 responses were included from participants who completed the survey up to part two and part three, respectively, leading to a total of 659 responses. The respondents had a mean age of 50.2 ± 12.1 years. 94.2% of them were GPs, 3.0% nurses, 2.1% allied healthcare professionals and 0.6% scientists/occupational physicians. Responses were collected from 18 different European countries (Figure 1), with the majority from Eastern Europe (36.1%), followed by Western Europe (33.4%), Southern Europe (12.1%), Northern Europe (10.0%) and United Kingdom & Ireland (8.3%). It is important to note that Eastern Europe is mainly represented by Romania (87.4% of Eastern European respondents). Results for the subregions separately can be found in the Supplementary Tables.

### 3.2. Overall perceptions concerning AF screening and screening approaches in general

#### 3.2.1. Presence of and perceived need for screening approaches for AF and other conditions

**Figure 2:**
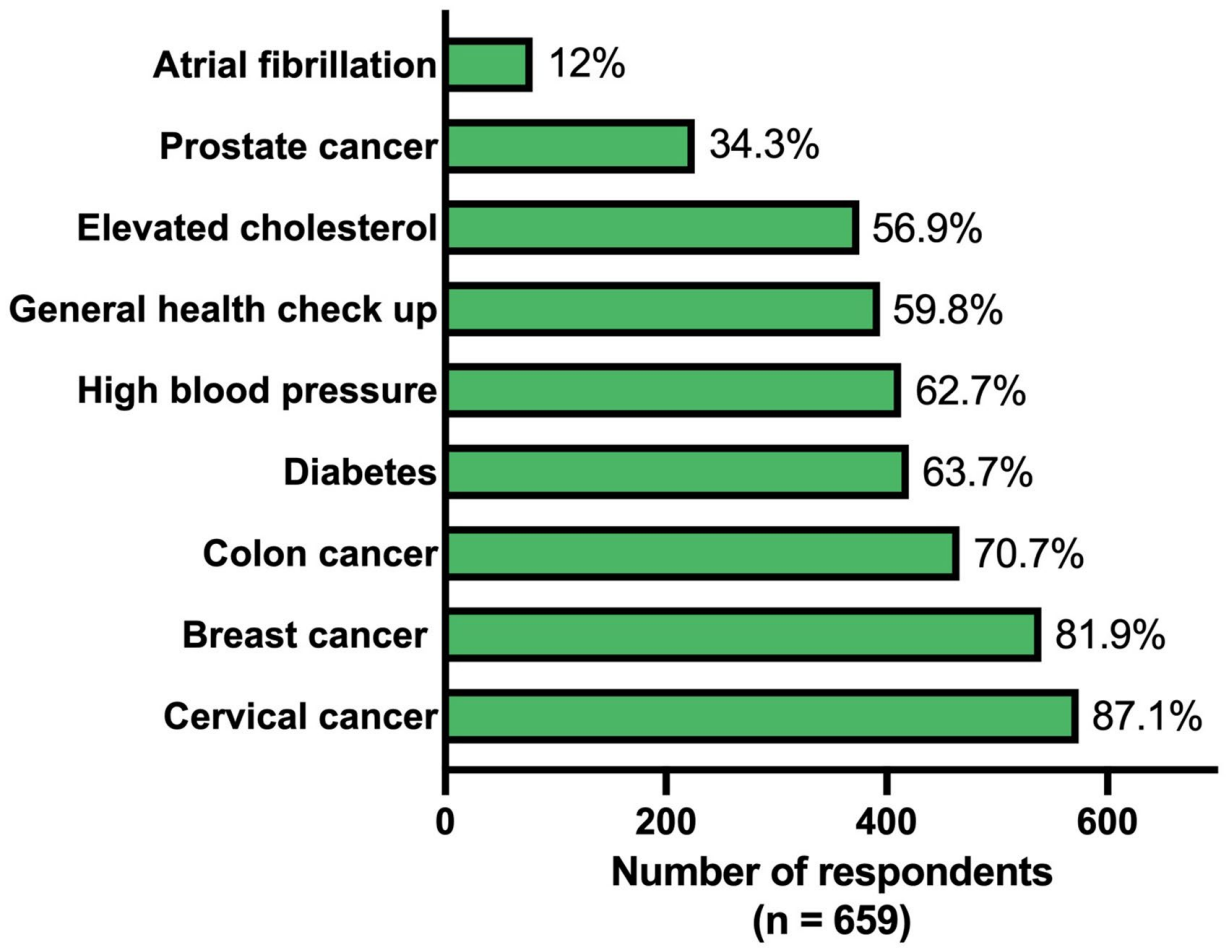
Availability of a standardized screening approach for different conditions.

Of all respondents, 12% indicated that an AF screening program was established in their region. Standardized screening was more often implemented for other conditions such as colon (70.7%), breast (81.9%) and cervical (87.1%) cancers (Figure 2). The presence of various screening approaches, including AF screening (*p* = 0.019), differed between the European regions (Supplementary Table 1). In both Southern Europe and United Kingdom & Ireland, 20.0% indicated that they applied some form of AF screening, while this was less in other regions (7.6–11.8%).

**Figure 3:**
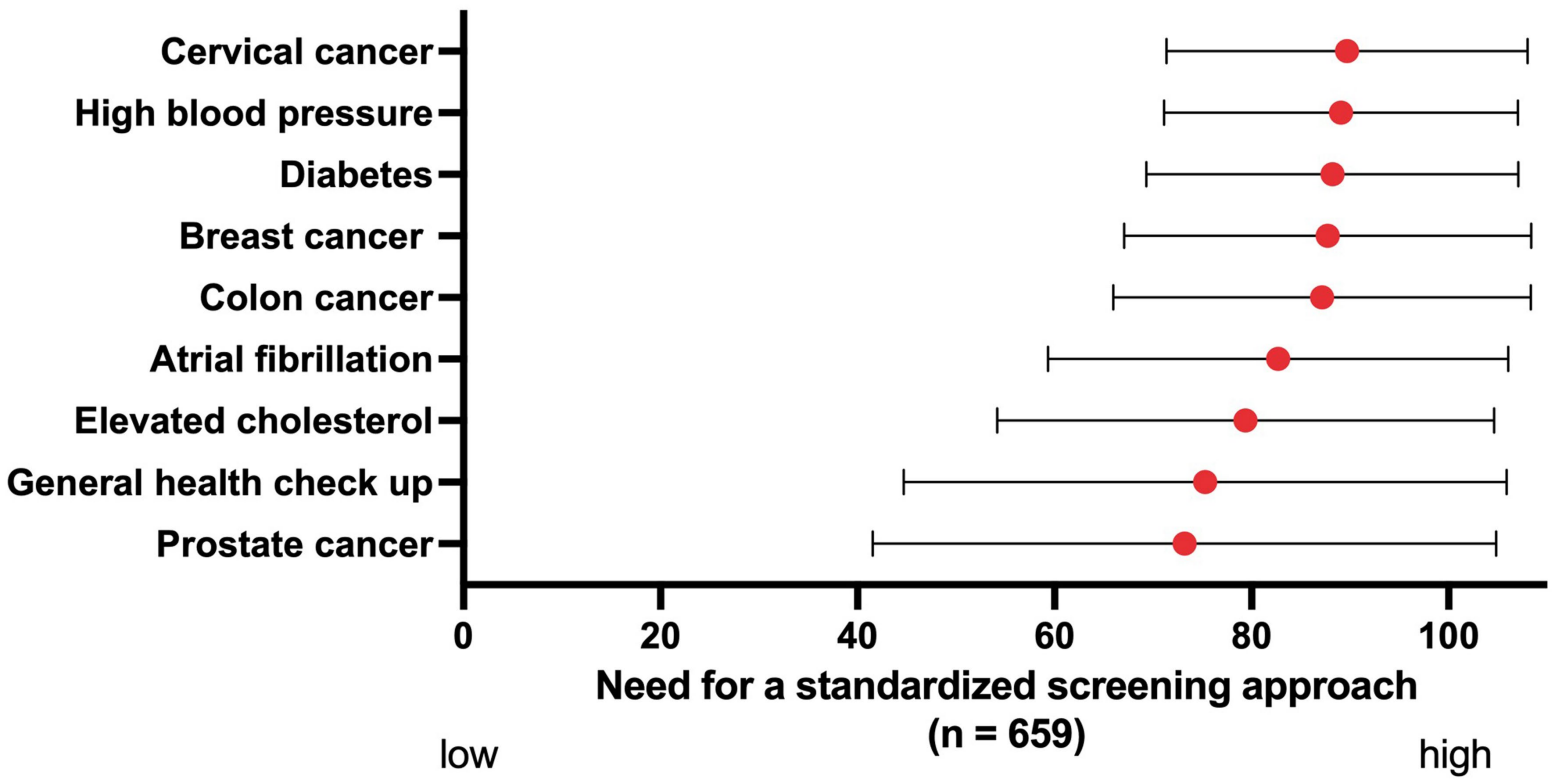
The perceived need for a standardized screening approach for different conditions, ranked on a scale from 0 to 100. Red dots: mean. Black bars: standard deviation.

The need for standardized AF screening was rated as 82.7 on a scale from 0 to 100, which was higher than for prostate cancer (73.2), a general health check-up (75.3) and elevated cholesterol (79.4), and lower than for colon cancer (87.2), breast cancer (87.7), diabetes (88.2), high blood pressure (89.1) and cervical cancer (89.7) (Figure 3). There was less variation within Eastern Europe compared to the other regions, with this region attaching great importance to screening for all mentioned conditions (Supplementary Table 2).

#### 3.2.2. Availability of ECG devices

Almost three in four respondents (72.1%) had a 12-lead ECG device available in their practice, while a single-lead ECG device was less common (10.8%). In addition, 6.5% had a 3-lead device, 3.2% had another ECG device (e.g., 15-lead, 6-lead) and 19.1% had no ECG device available. Notable regional differences were found between the availability of a 12-lead device (*p* < 0.001) and a single-lead device (*p* < 0.001) (Supplementary Table 3). The majority of GPs had a 12-lead ECG device in Northern Europe (98.5%), United Kingdom & Ireland (92.7%) and Western Europe (81.4%), while the availability of such a device was lowest in Southern (65.0%) and Eastern (53.8%) Europe. A single-lead device was fairly common in United Kingdom & Ireland (63.6%), but far less prevalent in other parts of Europe (1.5–7.1%).

#### 3.2.3. Regular health checks in patients over 65 years old

**Figure 4:**
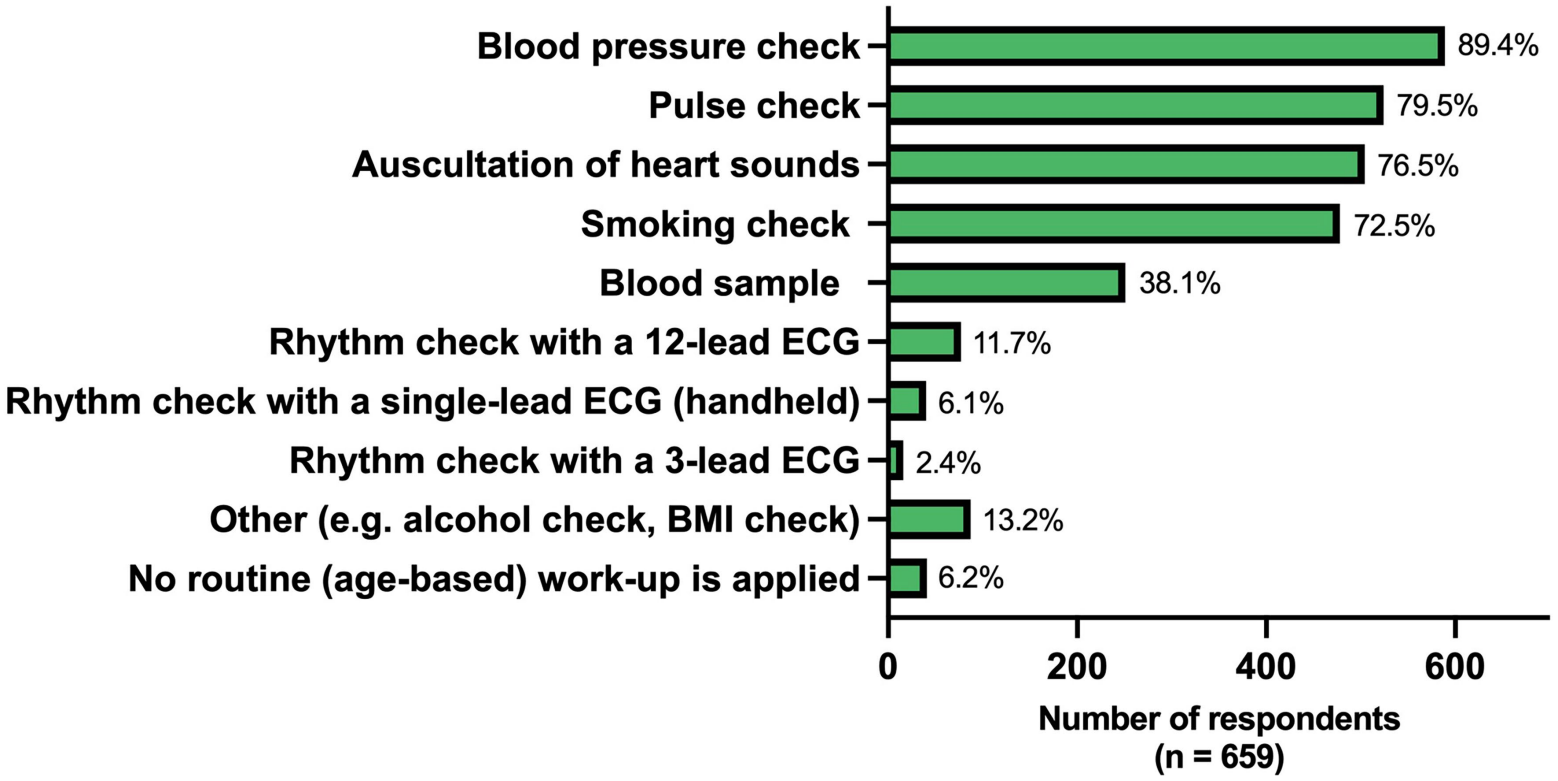
Regular health checks performed in patients ≥65 years coming for an outpatient visit. ECG, electrocardiogram; BMI, body mass index.

When asked which general health checks the GPs would do if a patient ≥65 years comes to their practice, blood pressure check (89.4%) and pulse check (79.5%) were most frequently performed. In contrast, only 11.7 and 6.1% would perform a rhythm check with a 12-lead ECG and a single-lead ECG device, respectively (Figure 4). A check with a single-lead device was carried out significantly different in the European regions (*p* < 0.001) and more often in United Kingdom & Ireland (36.4%) compared to the other regions (0–4.6%) (Supplementary Table 4).

### 3.3. Feasibility of opportunistic single-time point AF screening with a single-lead ECG device

Three in five respondents (59.3%) indicated that they would feel fairly or completely confident to rule out AF on a 30 s single-lead ECG strip. Others felt either somewhat confident (19.6%), slightly confident (9.0%) or not confident (12.1%). The confidence level was highest in United Kingdom & Ireland, where 85.4% were completely or fairly confident, followed by 76.9% in Southern, 60.6% in Northern, 58.2% in Western and 47.6% in Eastern Europe (*p*-values for fairly and completely confident were 0.010 and < 0.001, respectively) (Supplementary Table 5).

Overall, 28.7% of GPs indicated that more education on ECG and smarter ECG devices would improve their confidence. Moreover, 25.2% perceived a tele-healthcare service for upload of ECG tracings and rapid advice as helpful, and 23.4% would consider a standardized follow-up pathway with the possibility of a quick referral to the cardiologist of added value. The preferred assistance for each region can be found in Supplementary Table 6. About one GP in five (18.0%) indicated they did not need external help to rule out AF on a single-lead ECG tracing.

### 3.4. Requirements and barriers for the implementation of opportunistic single-time point AF screening with a single-lead ECG device

One third (32.2%) indicated that they could easily start conducting AF screening or have already implemented it. For others, barriers to implementing opportunistic single-time point AF screening with a single-lead device were diverse (Table 1). The most important ones were lack of education (18.2%), and insufficient (qualified) staff and ECG devices (18.5%). The need for more education varied per region (*p* < 0.001) and was the highest in Eastern Europe (29.0%) and lowest in United Kingdom & Ireland (3.8%) (Supplementary Table 7).

**Table 1:** Main obstacles for implementing opportunistic single-lead ECG screening for atrial fibrillation in patients ≥ 65 years or patients at risk of atrial fibrillation, even if it would be reimbursed.

	Respondents (*n* = 596)
There are insufficient resources to perform AF screening in my practice (i.e., personnel, ECG devices)	18.5%
I would need more education before undertaking AF screening	18.2%
I am concerned about detecting false positives that could lead to anxiety/harm to patients	10.3%
I am not confident about commencing treatment once AF has been diagnosed	7.0%
None, I can easily start conducting AF screening	26.0%
None, I have already implemented AF screening	6.2%
Other (e.g., lack of evidence benefits AF screening, lack of time, administration issues)	13.8%

The different responses were considered in relation to the total number of indicated reasons and presented as percentages. AF, atrial fibrillation; ECG, electrocardiogram.

The preferred strategies to overcome obstacles were to integrate AF screening with other healthcare programs (24.9%) and to set up software systems with algorithms able to identify patients most suitable for AF screening (24.3%) (Table 2). These two options were preferred by all different regions with *p*-values of 0.054 and 0.076, respectively (Supplementary Table 8).

**Table 2:** Suggested strategies to overcome obstacles concerning opportunistic single-lead ECG screening for atrial fibrillation.

	Respondents (*n* = 580)
AF screening could be integrated with other programs (e.g., flu vaccination, cancer screening)	24.9%
Integrated primary care software systems with algorithms to identify patients suitable for AF screening based on their age and/or medical history	24.3%
Integrating advice pathways from experts to GPs on how to interpret ECGs and prescribe appropriately	15.5%
Providing a structured way of analyzes of the tracings *via* a telehealth center	11.1%
Provision of patient leaflets or other information to increase patient education	10.7%
Additional settings (e.g., pharmacists or other healthcare professions) could be involved in screening	6.9%
Other (e.g., distribution of validated single-lead devices, more evidence and education, decision support tools, better time management)	6.8%

The different responses were considered in relation to the total number of indicated reasons and presented as percentages. AF, atrial fibrillation; ECG, electrocardiogram; GP, general practitioner.

Three out of five GPs (63.1%) indicated that opportunistic prolonged AF screening, in which at-risk patients wear an ECG patch for 2 weeks, would also be possible in their current practice, while 17.8% stated it would not be possible and 19.1% did not know (Supplementary Table 9).

## 4. Discussion

In this quantitative survey, GPs shared their views on the implementation of opportunistic single-time point AF screening, focusing on the feasibility, requirements and barriers.

The vast majority of GPs indicated that no AF screening program was available in their region. This is in line with the earlier qualitative study conducted in the framework of AFFECT-EU, in which healthcare professionals and regulators from 11 European countries confirmed that there are no systematic AF screening programs (6). Despite the ESC recommendation to conduct opportunistic AF screening by pulse taking or ECG rhythm strip in individuals ≥65 years (5), the US Preventive Services Task Force argues against implementation due to insufficient evidence on effectiveness, cost-effectiveness and potential harms (7). This lack of evidence was also indicated as a barrier by GPs in our current study. Questions remain about the advantages of AF detected by screening compared to routine care, in terms of stroke prevention and the benefits of anticoagulation (8). Data from recent studies, such as STROKESTOP and LOOP (9–11), were inconsistent, likely due to complex interactions between patient characteristics and screening strategies. However, in a current random effects model, AF screening was associated with a reduction of stoke compared to no screening (12). Several studies will report stoke outcome data in the near future, which will provide guidance for decision making.

In addition to general screening studies, research on the effectiveness of opportunistic screening specifically is also being conducted. The AFFORD study in Denmark concluded that opportunistic screening in primary care is feasible and results in the detection of new AF in 1% of patients (13). A similar study found a five-fold higher diagnostic yield of opportunistic screening compared to usual care (14). However, in the VITAL-AF study in the United States, screening for AF using a single-lead ECG at primary care visits did not affect new AF diagnoses among individuals aged 65 years or older compared with usual care (1.72% vs. 1.59%) (15). Similarly, a randomized controlled trial in the Netherlands failed to show a significant difference in new AF diagnoses (1.62% in the screening group versus 1.53% in usual care) (3). In several countries, usual care, irrespective of the methods used, already seems to be able to detect AF, making it more difficult to increase AF detection numbers by opportunistic screening (6). Despite the lack of evidence, GPs appear to recognize the need for AF screening programs almost as high as for various cancer screens, likely due to the devastating impact that stroke as the result of undetected AF might have.

Interestingly, three in five GPs (59.3%) feel fairly or completely confident to rule out AF on a 30 s single-lead ECG strip and 18% would not need any additional assistance to perform it. It is important to bear in mind the difference between ruling out AF and diagnosing AF. A study in which 49 GPs reviewed ECG tracings from 2,595 patients ≥65 years who were screened for AF as part of the SAFE study, concluded that most GPs could not accurately detect AF on either a 12-lead or a single-lead ECG. Therefore, the diagnosis of AF should be made by appropriately trained people (16). Nevertheless, these results are in contrast to those of Somerville et al. who found that GPs could accurately detect AF on a 12-lead ECG tracing (17).

Three out of ten GPs indicated that they could easily start or have already started opportunistic single-time point screening. Hence, the barriers perceived as most important (lack of education, insufficient staff and ECG devices), seem to be surmountable. To facilitate the effective implementation, GPs preferred two approaches of which the first was integration of AF screening with other healthcare programs. In the study by Engler et al., regulators and healthcare professionals indicated that it is not feasible to integrate AF screening with cancer screening programs due to the different target group and screening approach (6). Integration with flu vaccination does seem to be more appropriate. Secondly, GPs would consider an integrated primary care software system with an algorithm able to identify patients suitable for AF screening based on their age and/or medical history. At this point in time, traditional risk factor sets and scoring schemes such as the CHARGE-AF score are rather limited in their predictive ability and have limited user-friendliness as they often require electrocardiographic or laboratory parameters (18). Recently, Schnabel et al. developed a model based on 13 predictors that can robustly identify patients at risk of AF using machine learning techniques. This model could be implemented in automated screening tools to identify high-risk populations in primary care (19).

This study reported that three out of five GPs have the capacity to perform ECG patch screening in high-risk patients. Although prolonged patch use is often considered expensive and difficult (6), it seems a feasible approach for GPs. Moreover, previous studies like mSToPS, have already communicated that patch-based continuous ECG screening is feasible in primary care and able to identify AF (20, 21). Also, the large-scale GUARD-AF study (NCT04126486) is currently ongoing and will provide important information on the cost-effectiveness of patch-based AF screening, informing future studies and guidelines (22).

### 4.1. Regional differences

This study collected responses from 18 European countries, allowing us to examine regional differences. The most striking finding was that in United Kingdom & Ireland, some important steps appear to have been taken towards implementation of AF screening. For instance, 63.6% of GPs already have a single-lead ECG device available; 36.4% used these devices during regular health checks in patients ≥65 years; the GPs felt most confident to rule out AF on a rhythm strip; and there was the least need for additional education. In the United Kingdom, the National Health Service (NHS), are promoting NHS Health Checks for adults aged 40 to 74 in which GPs assess patients’ heart rhythms and determine whether AF is present if irregular. In addition, over 1,200 digital single-lead ECG devices have been delivered to GP practices in the United Kingdom to support opportunistic screening of undiagnosed AF (23).

The availability of a 12-lead ECG device in Southern and Eastern Europe was significantly lower than in the rest of Europe, possibly due to the difference in total wealth and spending on health. In addition, Eastern Europe showed the greatest need for additional education and attached great importance to screening not only for AF but for almost all proposed conditions. This suggests a certain gap between the AF and general health policies in Eastern Europe and United Kingdom & Ireland (24). However, it is important not to generalize these results for the whole of Eastern Europe, as Eastern Europe is mainly represented by Romania in this survey (87.4% of Eastern European respondents). To accommodate healthcare differences, e.g., differences in numbers of GPs per head of population, the differing roles of GPs within national healthcare systems and the time available to GPs per patient; and to set up national action plans and screening policies, joint actions of ministries of health, governmental bodies, healthcare professionals, clinical researchers and device industries will be required.

### 4.2. Limitations

The accessibility of GPs was considerably different in various European countries. In countries such as Romania, France and Belgium, GPs could be reached easily and efficiently by contacting them personally *via* a mailing list of local GPs. In other countries, e.g., Germany and Italy, it proved more difficult to reach GPs, due to the lack or minimal effect of such an up-to-date active mailing list. This may have led to some bias, and the situation in certain regions may be underrepresented in these survey results. As the survey was distributed through various GP organizations and personal GP networks, it is not possible to verify to how many people the survey was finally distributed to and no response rate can be determined. GPs with strong opinions on screening may be more inclined to participate in the survey, therefore some selection bias is possible.

## 5. Conclusion

GPs across Europe indicate a strong perceived need for a standardized AF screening approach, which is currently absent in most of Europe. In general, the perception of GPs concerning the implementation of opportunistic single time point AF screening with a single lead ECG device was positive. Most GPs would already feel confident in ruling out AF based on a single-lead ECG strip. The identified obstacles, such as lack of education, evidence, and resources, appear to be addressable. Finally, this study identified concrete suggestions for implementing of AF screening in clinical practice, namely integration with other healthcare programs and software systems with algorithms to identify patients most suitable for screening.

## Data availability statement

The raw data supporting the conclusions of this article will be made available by the authors, without undue reservation.

## Ethics statement

This study, involving human participants, was reviewed and approved by Ethik-Kommission der Ärztekammer Hamburg. The participants provided their written informed consent to participate in this study.

## Author contributions

LD, DE, RS, HH, CH, LN, BF, HW, MH, JL, and KG contributed to conception of the study and to design and review of the survey. MG, J-CD, CB, EP, GM, MF, MB, HP, CH, DE, RS, HH, LD, and PV contributed to the dissemination, promotion and/or translation of the survey. PV organized the database and performed the statistical analysis and wrote the first draft of the manuscript. All authors contributed to the article and approved the submitted version.

## Funding

The AFFECT-EU project has received funding from the European Research Council under the European Union’s Horizon 2020 research and innovation program under the grant agreement no. 847770.

## Conflict of interest

BF has received grants to the institution for investigator-initiated studies from the BMS-Pfizer Alliance, personal fees and nonfinancial support from BMS-Pfizer Alliance and Pfizer, and loan devices from AliveCor.

HH did receive personal lecture and consultancy fees from Abbott, Biotronik, Daiichi-Sankyo, Pfizer-BMS, Medscape, and Springer Healthcare Ltd. He received unconditional research grants through the University of Antwerp and/or the University of Hasselt from Abbott, Bayer, Biotronik, Biosense-Webster, Boston-Scientific, Boehringer-Ingelheim, Daicchi-Sankyo, Fibricheck/Qompium, Medtronic, and Pfizer-BMS.

HP received speaker fees from Bayer, Pfizer, Boehringer Ingelheim and Daiichi-Sankyo.

LN and CH hold an investigator initiated grant from Daiichi Sankyo (£75,000).

MF received speaker fees from Pfizer Poland and Boehringer Ingelheim Poland.

RS has received funding from the European Research Council (ERC) under the European Union’s Horizon 2020 research and innovation program under the grant agreement No 648131, from the European Union’s Horizon 2020 research and innovation program under the grant agreement No 847770 (AFFECT-EU) and German Center for Cardiovascular Research (DZHK e.V.) (81Z1710103 and 81Z0710114); German Ministry of Research and Education (BMBF 01ZX1408A) and ERACoSysMed3 (031L0239). RS has received lecture fees and advisory board fees from BMS/Pfizer outside this work.

HW was employed by Pfizer Pharma GmbH, Berlin, Germany.

MH was employed by StopAfib.org, Dallas, TX, United States.

The remaining authors declare that the research was conducted in the absence of any commercial or financial relationships that could be construed as a potential conflict of interest.

## Publisher’s note

All claims expressed in this article are solely those of the authors and do not necessarily represent those of their affiliated organizations, or those of the publisher, the editors and the reviewers. Any product that may be evaluated in this article, or claim that may be made by its manufacturer, is not guaranteed or endorsed by the publisher.

## References

[1] Gruwez H Evens S Proesmans T Duncker D Linz D Heidbuchel H. Accuracy of physicians interpreting Photoplethysmography and electrocardiography tracings to detect atrial fibrillation: INTERPRET-AF. Front Cardiovasc Med. (2021) 8:734737. doi: 10.3389/fcvm.2021.734737, PMID: 34616786 PMC8488290

[2] Nesheiwat Z Goyal A Jagtap M Shammas A. Atrial fibrillation (nursing). Treasure Island (FL): StatPearls (2022). 33760478

[3] Uittenbogaart SB Becker SJ Hoogsteyns M van Weert HC Lucassen WA. Experiences with screening for atrial fibrillation: a qualitative study in general practice. BJGP Open. (2022) 6:BJGPO.2021.0126. doi: 10.3399/BJGPO.2021.0126 34853006 PMC8958756

[4] Freedman B Camm J Calkins H Healey JS Rosenqvist M Wang J. Screening for atrial fibrillation: a report of the AF-SCREEN international collaboration. Circulation. (2017) 135:1851–67. doi: 10.1161/CIRCULATIONAHA.116.026693 28483832

[5] Hindricks G Potpara T Dagres N Arbelo E Bax JJ Blomstrom-Lundqvist C. 2020 ESC guidelines for the diagnosis and management of atrial fibrillation developed in collaboration with the European Association for Cardio-Thoracic Surgery (EACTS): the task force for the diagnosis and management of atrial fibrillation of the European Society of Cardiology (ESC) developed with the special contribution of the European heart rhythm association (EHRA) of the ESC. Eur Heart J. (2021) 42:373–498. doi: 10.1093/eurheartj/ehaa612, PMID: 32860505

[6] Engler D Hanson CL Desteghe L Boriani G Diederichsen SZ Freedman B. Feasible approaches and implementation challenges to atrial fibrillation screening: a qualitative study of stakeholder views in 11 European countries. BMJ Open. (2022) 12:e059156. doi: 10.1136/bmjopen-2021-059156, PMID: 35728895 PMC9214372

[7] Davidson KW Mangione C Ogedegbe G. US preventive services task force recommendation statement on screening for atrial fibrillation-reply. JAMA. (2022) 327:2022. doi: 10.1001/jama.2022.5207, PMID: 35608587

[8] Jones NR Taylor CJ Hobbs FDR Bowman L Casadei B. Screening for atrial fibrillation: a call for evidence. Eur Heart J. (2020) 41:1075–85. doi: 10.1093/eurheartj/ehz834, PMID: 31811716 PMC7060457

[9] Svennberg E Friberg L Frykman V Al-Khalili F Engdahl J Rosenqvist M. Clinical outcomes in systematic screening for atrial fibrillation (STROKESTOP): a multicentre, parallel group, unmasked, randomised controlled trial. Lancet. (2021) 398:1498–506. doi: 10.1016/S0140-6736(21)01637-8, PMID: 34469764

[10] Svendsen JH Diederichsen SZ Hojberg S Krieger DW Graff C Kronborg C. Implantable loop recorder detection of atrial fibrillation to prevent stroke (the LOOP study): a randomised controlled trial. Lancet. (2021) 398:1507–16. doi: 10.1016/S0140-6736(21)01698-6, PMID: 34469766

[11] Hoare S Powell A Modi RN Armstrong N Griffin SJ Mant J. Why do people take part in atrial fibrillation screening? Qualitative interview study in English primary care. BMJ Open. (2022) 12:e051703. doi: 10.1136/bmjopen-2021-051703, PMID: 35296474 PMC8928318

[12] McIntyre WF Diederichsen SZ Freedman B Schnabel RB Svennberg E Healey JS. Screening for atrial fibrillation to prevent stroke: a meta-analysis. Eur Heart J Open. (2022) 2:oeac044. doi: 10.1093/ehjopen/oeac044, PMID: 35919582 PMC9305505

[13] Hald J Poulsen PB Qvist I Holm L Wedell-Wedellsborg D Dybro L. Opportunistic screening for atrial fibrillation in a real-life setting in general practice in Denmark-the atrial fibrillation found on routine detection (AFFORD) non-interventional study. PLoS One. (2017) 12:e0188086. doi: 10.1371/journal.pone.0188086, PMID: 29131836 PMC5683635

[14] Zwart LA Jansen RW Ruiter JH Germans T Simsek S Hemels ME. Opportunistic screening for atrial fibrillation with a single lead device in geriatric patients. J Geriatr Cardiol. (2020) 17:149–54. doi: 10.11909/j.issn.1671-5411.2020.03.007, PMID: 32280331 PMC7118016

[15] Lubitz SA Atlas SJ Ashburner JM Lipsanopoulos ATT Borowsky LH Guan W. Screening for atrial fibrillation in older adults at primary care visits: VITAL-AF randomized controlled trial. Circulation. (2022) 145:946–54. doi: 10.1161/CIRCULATIONAHA.121.057014, PMID: 35232217 PMC8960369

[16] Mant J Fitzmaurice DA Hobbs FD Jowett S Murray ET Holder R. Accuracy of diagnosing atrial fibrillation on electrocardiogram by primary care practitioners and interpretative diagnostic software: analysis of data from screening for atrial fibrillation in the elderly (SAFE) trial. BMJ. (2007) 335:380. doi: 10.1136/bmj.39227.551713.AE, PMID: 17604299 PMC1952490

[17] Somerville S Somerville J Croft P Lewis M. Atrial fibrillation: a comparison of methods to identify cases in general practice. Br J Gen Pract. (2000) 50:727–9. PMCID: PMC1313802, PMID: 11050790 PMC1313802

[18] Alonso A Krijthe BP Aspelund T Stepas KA Pencina MJ Moser CB. Simple risk model predicts incidence of atrial fibrillation in a racially and geographically diverse population: the CHARGE-AF consortium. J Am Heart Assoc. (2013) 2:e000102. doi: 10.1161/JAHA.112.000102, PMID: 23537808 PMC3647274

[19] Schnabel RB Witt H Walker J Ludwig M Geelhoed B Kossack N. Machine learning-based identification of risk-factor signatures for undiagnosed atrial fibrillation in primary prevention and post-stroke in clinical practice. Eur Heart J Qual Care Clin Outcomes. (2022) 9:16–23. doi: 10.1093/ehjqcco/qcac013, PMID: 35436783 PMC9745664

[20] Steinhubl SR Waalen J Edwards AM Ariniello LM Mehta RR Ebner GS. Effect of a home-based wearable continuous ECG monitoring patch on detection of undiagnosed atrial fibrillation: the mSToPS randomized clinical trial. JAMA. (2018) 320:146–55. doi: 10.1001/jama.2018.8102, PMID: 29998336 PMC6583518

[21] Gladstone DJ Wachter R Schmalstieg-Bahr K Quinn FR Hummers E Ivers N. Screening for atrial fibrillation in the older population: a randomized clinical trial. JAMA Cardiol. (2021) 6:558–67. doi: 10.1001/jamacardio.2021.0038, PMID: 33625468 PMC7905702

[22] Singer DE Atlas SJ Go AS Lopes RD Lubitz SA McManus DD. ReducinG stroke by screening for UndiAgnosed atRial fibrillation in elderly inDividuals (GUARD-AF): rationale and design of the GUARD-AF randomized trial of screening for atrial fibrillation with a 14-day patch-based continuous ECG monitor. Am Heart J. (2022) 249:76–85. doi: 10.1016/j.ahj.2022.04.005, PMID: 35472303

[23] Wessex Academic Health Science Network. Independent evaluation of the AHSN network mobile ECG rollout program: Full report. (2019). London, UK: The National Health Service (NHS).

[24] Movsisyan NK Vinciguerra M Medina-Inojosa JR Lopez-Jimenez F. Cardiovascular diseases in central and Eastern Europe: a call for more surveillance and evidence-based health promotion. Ann Glob Health. (2020) 86:21. doi: 10.5334/aogh.2713, PMID: 32166066 PMC7059421

