# Supplementary material for: General practitioners’ perceptions on opportunistic single-time point screening for atrial fibrillation: A European quantitative survey
Source: Front Cardiovasc Med. 2023 Feb 15;10:1112561. doi: 10.3389/fcvm.2023.1112561 (PMC9975716; doi:10.3389/fcvm.2023.1112561)
Supplement: Supplementary file 1 [file Data_Sheet_1.docx]

Supplementary Material

General practitioners’ perceptions on opportunistic single-time point screening for atrial fibrillation: a European quantitative survey

**Paulien Vermunicht^*^, Mihaela Grecu, Jean-Claude Deharo, Claire M. Buckley, Elena Palà, Georges H. Mairesse, Michal M. Farkowski, Marco Bergonti, Helmut Pürerfellner, Coral L. Hanson, Lis Neubeck, Ben Freedman, Henning Witt, Mellanie T. Hills, Jenny Lund, Katrina Giskes, Daniel Engler, Renate B. Schnabel, Hein Heidbuchel, Lien Desteghe, for the AFFECT-EU investigators**

*** Correspondence:** Paulien Vermunicht: Paulien.Vermunicht@uantwerpen.be

# Supplementary annexes

## Supplementary annex 1: Survey on primary health care professionals’ perceptions of opportunistic screening for atrial fibrillation

Dear colleague,

Atrial fibrillation (AF) is often asymptomatic and therefore unrecognized in our patients, who are nevertheless at increased risk of stroke. The aim of this survey is to evaluate perceptions about AF screening.

You/Your practice are/is invited to participate in a short online survey, which will take 5-7 minutes of your time.

When we refer to ‘opportunistic* AF screening’ we mainly refer to the use of a handheld device such as the one below in patient ≥ 65 or patients at AF risk, unless otherwise indicated.


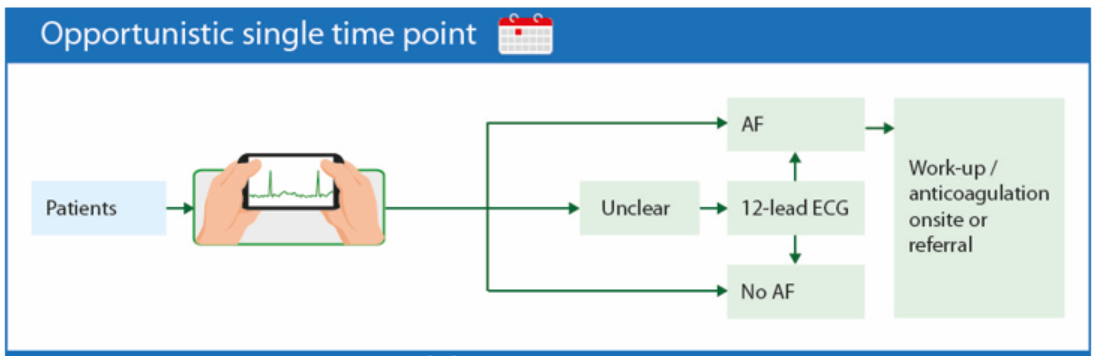


* Opportunistic screening: health care professional specifically checks for AF during routine consultations for other reasons.

Your participation would be greatly appreciated!

If you have any concerns or questions, please do not hesitate to contact:

- Daniel Engler: d.engler@uke.de, University Heart and Vascular Center Hamburg Eppendorf
- Lien Desteghe: lien.desteghe@uantwerpen.be, Antwerp University | Antwerp University Hospital

Responding to this survey is voluntary. The collected anonymized data will be kept for a maximum of 10 years. Data collected will not be transferred to any third party and handling of the anonymized data will be in accordance with the European Regulation 2016/679 with regards to data protection.

**Consent form:**

I agree that I have understood the above information.

I understand that once the survey is submitted it will not be possible to ascertain which data belongs to me and the researchers will be unable to delete these data.

Yes
 No

| 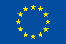 | AFFECT-EU receiving funding from the European Union’s Horizon 2020 research and innovation Programme under grant agreement N°847770. This reflects the authors’ view only. The Commission is not responsible for any use that may be made of the information it contains. |
| --- | --- |

**1. Personal information:**

- 1. **What is your profession?**

General practitioner

Allied healthcare professional

Nurse

Other: _________

- 1. **What is your gender?**

Male

Female

Transgender

Prefer not to say

- 1. **How old are you?**

_____ Years

- 1. **Country/region:**__________________

**2. Your perceptions concerning AF screening and current approaches to screening in general:**

- 1. **Is a standardized disease screening approach / disease programme (case detection) for the following conditions already implemented in your region (please tick as appropriate)?**

|  | **yes** | **no** |
| --- | --- | --- |
| Diabetes |  |  |
| Elevated cholesterol |  |  |
| Atrial fibrillation |  |  |
| High blood pressure |  |  |
| Colon cancer |  |  |
| Breast cancer |  |  |
| Prostate cancer |  |  |
| Cervical cancer |  |  |
| General health check-up |  |  |

- 1. **How would you rate the need for a standardized screening programme approach for the following conditions (irrespective of whether it is mandatory or not in your region) based on your clinical practice?**

| **Diabetes** | 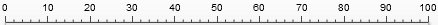  **Low High** |
| --- | --- |
| **Elevated cholesterol** | 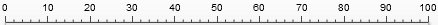 |
| **Atrial fibrillation** | 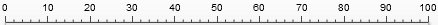 |
| **High blood pressure** | 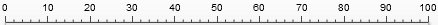 |
| **Colon cancer** | 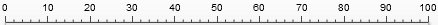 |
| **Breast cancer** | 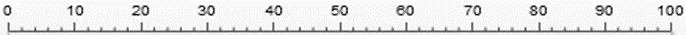 |
| **Prostate cancer** | 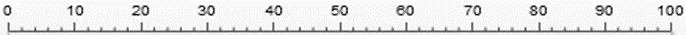 |
| **Cervical cancer** | 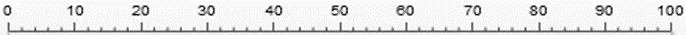 |
| **General health check up** | 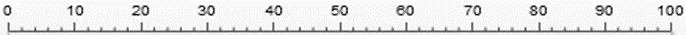 |

- 1. **Do you have an ECG device at your clinical practice?** (multiple answers possible)

12-lead ECG

3-lead ECG

Single-lead ECG (handheld)

Other:_______

No

- 1. **A patient ≥ 65 years comes to your practice for an outpatient visit (for whatever reason); do you take this opportunity to do any of the following checks (if time permits)?** (multiple answers possible as relevant to your practice)

Pulse check

Ausculation of heart sounds

Blood pressure check

Rhythm check with a 12-lead ECG

Rhythm check with a 3-lead ECG

Rhythm check with a single-lead ECG (handheld)

Blood sample (e.g. lipids, thyroid status etc.)

Smoking check

No routine (age-based) work-up is applied

Other: Specify_________________

**3. Feasibility of opportunistic single-lead ECG screening:**

1. **How confident are you to rule out atrial fibrillation (AF) rhythm present on this 30-second single-lead ECG rhythm strip such as shown below?**

not confident

slightly confident

somewhat confident

fairly confident

completely confident

| Lead 1 | 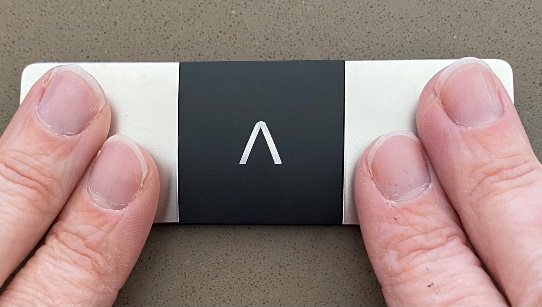 |
| --- | --- |
| Sinus rhythm | 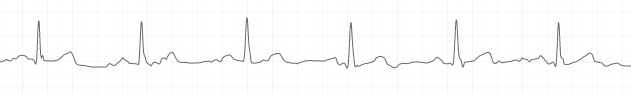 |
| Possible Atrial fibrillation | 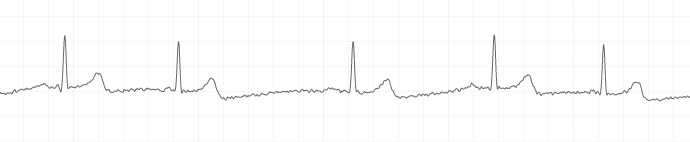 |

1. **What would assist in improving your confidence to rule out AF based on such kind of single-lead ECG (see above)?** (multiple answers possible)

More education on ECG in general and novel ECG devices in particular

Tele-healthcare service for upload of the ECG/ tracing and rapid advice within the same day

Standardized follow-up pathway and possibility for rapid referral to a cardiologist

Other procedures:___________________

Nothing; I am already confident in ruling out AF based on a single-lead ECG; no external help needed

**4. Implementation requirements and barriers**

- 1. **Please identify the TWO MAIN obstacles for implementing opportunistic single-lead ECG screening for patients over ≥ 65 years or patients at AF risk (as in the figure below) at the current stage in your practice (even if it would be reimbursed)**


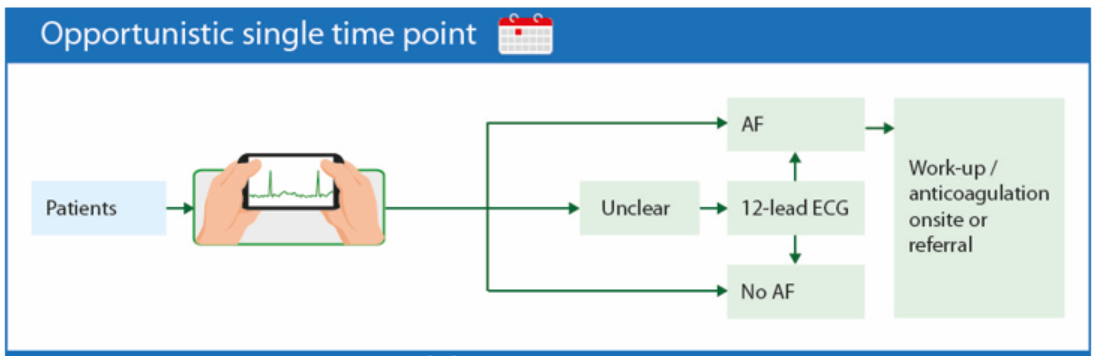


I would need more education before undertaking AF screening.

I am concerned about detecting false positives that could lead to anxiety/harm to patient.

I am not confident about commencing treatment once AF has been diagnosed.
 There are insufficient resources to perform AF screening in my practice (i.e. personnel, ECG qualification)

Other: specify____________

None, I can easily start conducting AF screening
 None, I have already implemented AF screening

- 1. **How could these obstacles be overcome to conduct AF screening in patients from your practice if reimbursed? Please choose TWO MAIN strategies that could be implemented.**

AF screening could be integrated with other programmes (e.g. flu vaccination, cancer screening)

Additional settings (e.g. pharmacists or other healthcare professions) could be involved in screening

Provision of patient leaflets or other information to increase patient education

Integrated primary care software systems with algorithms to identify patients suitable for AF screening based on their age and/or medical history

Providing a structured way of analyses of the tracings via a telehealth center.

Integrating advice pathways from experts to GP´s how to interpret ECGs and prescribe appropriately.

- 1. **Would your current practice have capacity to conduct ECG patch screening for 2 weeks for relevant patients e.g. 2-5 patients each week.** (Please see approach below)


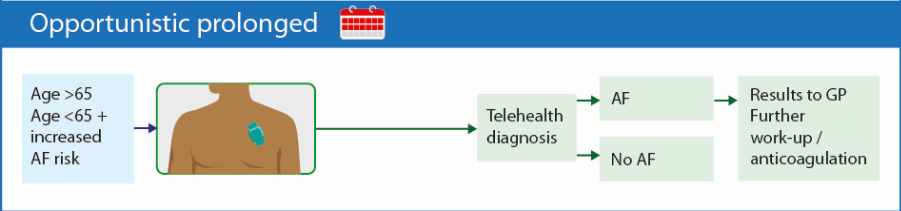


Yes

No

Do not know

- 1. **Do you have any further comments or suggestions about AF screening implementation in your practice?**

______________________________________________________________________________________________________________________________________________________________________________________________________________________________________________________________________________

## Supplementary annex 2: Validation of the survey

The survey was validated for its content and further refined during four consecutive phases. In the first phase, experts of the AFFECT-EU consortium involved in AF patient care (Ben Freedman, Coral L. Hanson, Daniel Engler, Hein Heidbuchel, Lis Neubeck, Renate B. Schnabel) reviewed the survey and provided specific feedback on the content considering five aspects: (i) is it a valid tool to answer our research questions?; (ii) does the survey address all the relevant issues or are there any obvious omissions?; (iii) is the order of the questions correct and is the flow logical?; (iv) is the text easy to read and comprehend?; and (v) is the questionnaire length appropriate? Secondly, four GPs were asked to provide feedback on the same five aspects. In the third phase, the content of the survey was discussed during a workshop with the AFFECT-EU consortium partners. During the final phase, three GPs reviewed the updated version of the survey and after their feedback, a final optimisation of the survey occurred.

# Supplementary tables

## Supplementary table 1: Availability of a standardised screening approach for different conditions, presented for different European regions.

| Condition | All respondents  (n = 659) | Eastern Europe  (n = 238) | Western Europe (n = 220) | Southern Europe (n = 80) | Northern Europe (n = 66) | UK & Ireland (n = 55) | P-value between regions |
| --- | --- | --- | --- | --- | --- | --- | --- |
| Atrial fibrillation | 79 (12.0) | 28 (11.8) | 19 (8.6) | 16 (20.0) | 5 (7.6) | 11 (20.0) | **0.019** |
| Prostate cancer | 226 (34.3) | 88 (37.0) | 91 (41.4) | 33 (41.3) | 5 (7.6) | 9 (16.4) | **<0.001** |
| Elevated cholesterol | 375 (56.9) | 135 (56.7) | 120 (54.5) | 63 (78.8) | 33 (50.0) | 24 (43.6) | **<0.001** |
| General health check up | 394 (59.8) | 157 (66.0) | 129 (58.6) | 54 (67.5) | 29 (43.9) | 25 (45.5) | **0.002** |
| High blood pressure | 413 (62.7) | 159 (66.8) | 124 (56.4) | 67 (83.8) | 35 (53.0) | 28 (50.9) | **<0.001** |
| Diabetes | 420 (63.7) | 142 (59.7) | 148 (67.3) | 62 (77.5) | 38 (57.6) | 30 (54.5) | **0.014** |
| Colon cancer | 466 (70.7) | 102 (42.9) | 214 (97.3) | 79 (98.8) | 19 (28.8) | 52 (94.5) | **<0.001** |
| Breast cancer | 540 (81.9) | 128 (53.8) | 217 (98.6) | 78 (97.5) | 66 (100.0) | 51 (92.7) | **<0.001** |
| Cervical cancer | 574 (87.1) | 185 (77.7) | 197 (89.5) | 75 (93.8) | 64 (97.0) | 53 (96.4) | **<0.001** |

Data are presented as absolute numbers and percentages of respondents who indicated a screening approach was present in their region. It is important to note that Eastern Europe is mainly represented by Romania (87.4% of Eastern European respondents).

## Supplementary table 2: Perceived need for a standardised screening approach for different conditions, rated on a scale from 0 to 100, presented for different European regions.

| Condition |  | All  respondents  (n = 659) | Eastern  Europe  (n = 238) | Western  Europe (n = 220) | Southern  Europe (n = 80) | Northern  Europe (n = 66) | UK &  Ireland (n = 55) | P-value between regions |
| --- | --- | --- | --- | --- | --- | --- | --- | --- |
| Atrial fibrillation | (1) (2) | 82.7 ± 23.4  93 (71 – 100) | 92.5 ± 16.7 100 (96 – 100) | 77.2 ± 24.9 83 (61 – 100) | 84.2 ± 20.5 90 (78.3 – 100) | 63.3 ± 26.3 63 (50 – 82.3) | 83.1 ± 21.5 90 (76 – 100) | **<0.001** |
| Prostate cancer | (1) (2) | 73.2 ± 31.6 86 (50 – 100) | 91.6 ± 18.0 100 (91.8 – 100) | 66.9 ± 31.7 77.5 (50 – 98) | 68.3 ± 32.3 78 (40 – 100) | 49.4 ± 33.3 50 (20 – 80.5) | 54.5 ± 33.4 57 (21 – 86) | **<0.001** |
| Elevated cholesterol | (1) (2) | 79.4 ± 25.2 89 (70 – 100) | 91.7 ± 17.4 100 (90 – 100) | 71.3 ± 27.0 78.5 (52 – 95) | 82.4 ± 21.8 92 (73 – 100) | 65.1 ± 28.4 70.5 (46.5 – 86.3) | 71.1 ± 22.9 75 (50 – 92) | **<0.001** |
| General health check-up | (1) (2) | 75.3 ± 30.6 100 (52 – 100) | 93.3 ± 17.6 100 (99.8 – 100) | 71.7 ± 29.6 80 (51 – 100) | 70.6 ± 30.4 80 (50 – 100) | 46.3 ± 31.2 50 (18.8 – 71.3) | 53.6 ± 31.0 50 (26 – 80) | **<0.001** |
| High blood pressure | (1) (2) | 89.1 ± 18.0 100 (83 – 100) | 96.2 ± 11.1 100 (100 – 100) | 85.7 ± 19.6 91.5 (80 – 100) | 90.3 ± 14.0 96.5 (86.3 – 100) | 77.2 ± 24.7 85 (61.8 – 100) | 84.2 ± 18.3 91 (72 – 100) | **<0.001** |
| Diabetes | (1) (2) | 88.2 ± 18.9 100 (81 – 100) | 94.3 ± 15.0 100 (98 – 100) | 85.9 ± 20.4  93 (80 – 100) | 89.5 ± 16.7 96 (82.3 – 100) | 76.2 ± 22.6 80 (61.8 – 97.8) | 83.5 ± 16.5 86 (75 – 100) | **<0.001** |
| Colon cancer | (1) (2) | 87.2 ± 21.2 100 (81 – 100) | 92.8 ± 16.2 100 (96.8 – 100) | 87.5 ± 20.8 98.5 (82 – 100) | 93.0 ± 14.2 100 (90.3 – 100) | 62.3 ± 26.6 60 (45.8 – 84.3) | 82.6 ± 21.1 90 (70 – 100) | **<0.001** |
| Breast cancer | (1) (2) | 87.7 ± 20.7 100 (81 – 100) | 94.9 ± 13.2 100 (100 – 100) | 84.2 ± 23.1 94.5 (77.3 – 100) | 92.3 ± 14.2 100 (90 – 100) | 76.0 ± 24.4 82 (60 – 100) | 78.1 ± 26.1 89 (60 – 100) | **<0.001** |
| Cervical cancer | (1) (2) | 89.7 ± 18.3 100 (85 – 100) | 94.7 ± 14.5 100 (100 – 100) | 87.1 ± 19.8 98 (80 – 100) | 92.2 ± 14.8 100 (90 – 100) | 81.5 ± 21.0 89.5 (69.8 – 100) | 84.3 ± 21.7 95 (75 – 100) | **<0.001** |

Data are presented as (1) mean ± standard deviation and as (2) median (Q1 – Q3). It is important to note that Eastern Europe is mainly represented by Romania (87.4% of Eastern European respondents).

## Supplementary table 3: The availability of different ECG devices, presented for different European regions.

| ECG device | All respondents  (n = 659) | Eastern Europe  (n = 238) | Western Europe (n = 220) | Southern Europe (n = 80) | Northern Europe (n = 66) | UK & Ireland (n = 55) | P-value between regions |
| --- | --- | --- | --- | --- | --- | --- | --- |
| 12-lead ECG | 475 (72.1) | 128 (53.8) | 179 (81.4) | 52 (65.0) | 65 (98.5) | 51 (92.7) | **<0.001** |
| 3-lead ECG | 43 (6.5) | 23 (9.7) | 10 (4.5) | 1 (1.3) | 2 (3.0) | 7 (12.7) | **0.008** |
| Single-lead ECG (handheld) | 71 (10.8) | 17 (7.1) | 14 (6.4) | 4 (5.0) | 1 (1.5) | 35 (63.6) | **<0.001** |
| Other | 21 (3.2) | 10 (4.2) | 5 (2.3) | 3 (3.8) | 1 (1.5) | 2 (3.6) | 0.716 |
| No | 126 (19.1) | 68 (28.6) | 33 (15.0) | 23 (28.7) | 0 (0) | 2 (3.6) | **<0.001** |

Data are presented as absolute numbers and percentages of respondents who indicated they had the ECG device available. It is important to note that Eastern Europe is mainly represented by Romania (87.4% of Eastern European respondents).

## Supplementary table 4: Overview of regular health checks the respondents would perform in a ≥ 65 years patients that comes to their practice for an outpatient visit, presented for different European regions.

| Health check | All  respondents  (n = 659) | Eastern  Europe  (n = 238) | Western  Europe (n = 220) | Southern Europe (n = 80) | Northern Europe (n = 66) | UK &  Ireland (n = 55) | P-value between regions |
| --- | --- | --- | --- | --- | --- | --- | --- |
| Blood pressure check | 589 (89.4) | 234 (98.3) | 197 (89.5) | 66 (82.5) | 44 (66.7) | 48 (87.3) | **<0.001** |
| Pulse check | 524 (79.5) | 227 (95.4) | 180 (81.8) | 45 (56.3) | 30 (45.5) | 42 (76.4) | **<0.001** |
| Auscultation of heart sounds | 504 (76.5) | 206 (86.6) | 178 (80.9) | 53 (66.3) | 45 (68.2) | 22 (40.0) | **<0.001** |
| Smoking check | 478 (72.5) | 183 (76.9) | 147 (66.8) | 60 (75.0) | 43 (65.2) | 45 (81.8) | **0.036** |
| Blood sample | 251 (38.1) | 47 (19.7) | 133 (60.5) | 30 (37.5) | 17 (25.8) | 24 (43.6) | **<0.001** |
| Rhythm check with a 12-lead ECG | 77 (11.7) | 43 (18.1) | 17 (7.7) | 9 (11.3) | 3 (4.5) | 5 (9.1) | **0.003** |
| Rhythm check with a single-lead ECG (handheld) | 40 (6.1) | 11 (4.6) | 8 (3.6) | 1 (1.3) | 0 (0) | 20 (36.4) | **<0.001** |
| Rhythm check with a 3-lead ECG | 16 (2.4) | 12 (5.0) | 3 (1.4) | 0 (0) | 0 (0) | 1 (1.8) | **0.020** |
| Other (e.g. alcohol check, BMI check) | 87 (13.2) | 27 (11.3) | 43 (19.5) | 7 (8.8) | 5 (7.6) | 5 (9.1) | **0.014** |
| No routine (age-based) work-up is applied | 41 (6.2) | 3 (1.3) | 14 (6.4) | 6 (7.5) | 14 (21.2) | 4 (7.3) | **<0.001** |

Data are presented as absolute numbers and percentages of respondents who would perform the health check. It is important to note that Eastern Europe is mainly represented by Romania (87.4% of Eastern European respondents).

## Supplementary table 5: Confidence level of respondents to rule out atrial fibrillation based on a 30-second single-lead ECG rhythm strip, presented for different European regions.

| Confidence level | All respondents  (n = 643) | Eastern Europe  (n = 231) | Western Europe (n = 213) | Southern Europe (n = 78) | Northern Europe (n = 66) | UK & Ireland (n = 55) | P-value between regions |
| --- | --- | --- | --- | --- | --- | --- | --- |
| Not confident | 78 (12.1) | 34 (14.7) | 30 (14.1) | 7 (9.0) | 5 (7.6) | 2 (3.6) | 0.093 |
| Slightly confident | 58 (9.0) | 25 (10.8) | 19 (8.9) | 3 (3.8) | 10 (15.2) | 1 (1.8) | **0.041** |
| Somewhat confident | 126 (19.6) | 62 (26.8) | 40 (18.8) | 8 (10.3) | 11 (16.7) | 5 (9.1) | **0.003** |
| Fairly confident | 270 (42.0) | 83 (35.9) | 84 (39.4) | 37 (47.4) | 38 (57.6) | 28 (50.9) | **0.010** |
| Completely confident | 111 (17.3) | 27 (11.7) | 40 (18.8) | 23 (29.5) | 2 (3.0) | 19 (34.5) | **<0.001** |

Data are presented as absolute numbers and percentages. It is important to note that Eastern Europe is mainly represented by Romania (87.4% of Eastern European respondents).

## Supplementary table 6: Preferred assistance to rule out atrial fibrillation based on a 30-second single-lead ECG rhythm strip, presented for different European regions.

|  | All Respondents (n=643) | Eastern Europe  (n = 231) | | Western Europe (n = 213) | | | Southern Europe (n = 78) | | Northern Europe (n = 66) | | UK &  Ireland (n = 55) | P-value between regions |
| --- | --- | --- | --- | --- | --- | --- | --- | --- | --- | --- | --- | --- |
| More education on ECG in general and novel ECG devices in particular | 28.7 | | 36.3 | | 20.4 | 29.1 | | 33.1 | | 23.3 | | **<0.001** |
| Tele-healthcare service for upload of the ECG tracing and rapid advice within the same day | 25.2 | | 20.7 | | 30.3 | 25.9 | | 21.7 | | 27.3 | | **0.002** |
| Standardised follow-up pathway and possibility for rapid referral to a cardiologist | 23.4 | | 29.6 | | 22.5 | 11.8 | | 23.2 | | 17.9 | | **<0.001** |
| Other procedures | 4.6 | | 1.7 | | 6.6 | 6.4 | | 8.3 | | 2.4 | | **0.005** |
| Nothing; I am already confident in ruling out AF based on a single-lead ECG; no external help needed | 18.0 | | 11.7 | | 20.2 | 26.9 | | 13.6 | | 29.1 | | **0.002** |

The different responses were considered in relation to the total number of indicated reasons and presented as percentages. ECG: electrocardiogram, AF: atrial fibrillation. It is important to note that Eastern Europe is mainly represented by Romania (87.4% of Eastern European respondents).

## Supplementary table 7: Main obstacles for implementing opportunistic single-lead ECG screening for atrial fibrillation in patients ≥ 65 years or patients at risk of atrial fibrillation, presented for different European regions.

|  | All respondents (n=596) | | Eastern Europe  (n = 207) | Western Europe (n = 205) | Southern Europe (n = 71) | Northern Europe (n = 61) | UK & Ireland (n = 52) | P-value between regions |
| --- | --- | --- | --- | --- | --- | --- | --- | --- |
| There are insufficient resources to perform AF screening in my practice (i.e. personnel, ECG devices) | | 18.5 | 18.8 | 12.2 | 26.4 | 32.0 | 17.3 | **0.004** |
| I would need more education before undertaking AF screening | | 18.2 | 29.0 | 14.4 | 12.7 | 13.1 | 3.8 | **<0.001** |
| I am concerned about detecting false positives that could lead to anxiety/harm to patients | | 10.3 | 12.1 | 7.8 | 9.2 | 13.9 | 10.6 | 0.204 |
| I am not confident about commencing treatment once AF has been diagnosed | | 7.0 | 3.9 | 13.2 | 4.2 | 1.6 | 4.8 | **<0.001** |
| None, I can easily start conducting AF screening | | 26.0 | 27.5 | 27.8 | 28.2 | 24.6 | 11.5 | 0.165 |
| None, I have already implemented AF screening | | 6.2 | 2.4 | 8.3 | 4.2 | 0.0 | 23.1 | **<0.001** |
| Other (e.g. lack of evidence benefits AF screening, lack of time, administration issues) | | 13.8 | 6.3 | 16.3 | 16.9 | 14.8 | 28.8 | **<0.001** |

The different responses were considered in relation to the total number of indicated reasons and presented as percentages. AF: atrial fibrillation, ECG: electrocardiogram. It is important to note that Eastern Europe is mainly represented by Romania (87.4% of Eastern European respondents).

## Supplementary table 8: Suggested strategies to overcome obstacles concerning opportunistic single-lead ECG screening for atrial fibrillation, presented for different European regions.

|  | All respondents (n=580) | | Eastern Europe  (n = 205) | Western Europe (n = 199) | Southern Europe (n = 69) | Northern Europe (n = 60) | UK & Ireland (n = 47) | P-value between regions |
| --- | --- | --- | --- | --- | --- | --- | --- | --- |
| AF screening could be integrated with other programmes (e.g. flu vaccination, cancer screening) | | 24.9 | 22.7 | 23.1 | 28.3 | 29.2 | 31.9 | 0.054 |
| Integrated primary care software systems with algorithms to identify patients suitable for AF screening based on their age and/or medical history | | 24.3 | 26.1 | 23.9 | 26.8 | 24.2 | 14.9 | 0.076 |
| Integrating advice pathways from experts to GPs how to interpret ECGs and prescribe appropriately | | 15.5 | 21.2 | 16.3 | 8.0 | 8.3 | 7.4 | **<0.001** |
| Providing a structured way of analyses of the tracings via a telehealth center | | 11.1 | 10.7 | 12.8 | 12.3 | 5.8 | 10.6 | 0.237 |
| Provision of patient leaflets or other information to increase patient education | | 10.7 | 13.7 | 11.1 | 6.5 | 5.0 | 9.6 | **0.018** |
| Additional settings (e.g. pharmacists or other healthcare professions) could be involved in screening | | 6.9 | 1.7 | 6.0 | 11.6 | 16.7 | 13.8 | **<0.001** |
| Other (e.g. distribution of validated single-lead devices, more evidence and education, decision support tools, better time management) | | 6.8 | 3.9 | 6.8 | 6.5 | 10.8 | 11.7 | **0.003** |

The different responses were considered in relation to the total number of indicated reasons and presented as percentages. AF: atrial fibrillation, ECG: electrocardiogram, GP: general practitioner. It is important to note that Eastern Europe is mainly represented by Romania (87.4% of Eastern European respondents).

## Supplementary table 9: The capacity of respondents to perform opportunistic prolonged atrial fibrillation screening, in which relevant patients wear an electrocardiogram patch for 2 weeks, presented for different European regions.

| Opportunistic prolonged screening possible | All respondents (n=596) | Eastern Europe  (n = 207) | Western Europe (n = 205) | Southern Europe (n = 71) | Northern Europe (n = 61) | UK & Ireland (n = 52) | P-value between regions |
| --- | --- | --- | --- | --- | --- | --- | --- |
| Yes | 376 (63.1) | 154 (74.4) | 127 (62.0) | 39 (54.9) | 22 (36.1) | 34 (65.4) | **<0.001** |
| No | 106 (17.8) | 20 (9.7) | 39 (19.0) | 17 (23.9) | 16 (26.2) | 14 (26.9) | **0.002** |
| Do not know | 114 (19.1) | 33 (15.9) | 39 (19.0) | 15 (21.1) | 23 (37.7) | 4 (7.7) | **<0.001** |

Data are presented as absolute numbers and percentages. It is important to note that Eastern Europe is mainly represented by Romania (87.4% of Eastern European respondents).
